# Dr. Roget's Reply to Mr. Hume

**Published:** 1812-12

**Authors:** P. M. Roget

**Affiliations:** Bernard-street, Russell-square


*To the Editors of the Medical and Physical Journal.*

GENTLEMEN,

IT is with reluctance that I again trouble you on a subject in which the public is so little interested as the controversy respecting the priority of Dr. Marcet or Mr. Hume's application of ammonia in the test for the detection of arsenic. But, as the latter gentleman still asserts his exclusive claim to the invention, in a way that impeaches the accuracy of my references, I beg leave, for the sake of those readers who would not be at the pains to consult the original documents, to correct a material error of which Mr. Hume has been guilty in quoting his own letter.

He infers, that he originally suggested the employment of ammonia with the nitrate of silver, from his having used the expression "*or any other alkali.*"* Now it will be found, that, in the only instance in which that expression occurs,† it is used with reference to a totally different test, namely, the *sulphate of copper*, and has, therefore, nothing to do with the subject in dispute, which relates entirely to the employment of ammonia with the *nitrate of silver*.

After this specimen of Mr. Hume's mode of reasoning, I trust it is unnecessary for me to pursue any farther a dis-

---

* His words are these: "The question seems capable of being reduced merely to this; What is comprehended under such expressions as *potass, lime, soda,* or *any other alkali?* Does not this language include *ammonia, barytes, strontites,* and *magnesia* at least?" See the Medical and Physical Journal for October last, p. 289.

† Medical and Physical Journal, vol, xxiii. p. 448, line 34.

cussion cussion in which I see no prospect of satisfying his expectations, and which must already have exhausted the patience of your readers.

I am, Gentlemen,

Your most obedient Servant,

*Bernard-street, Russell-square,* P. M. ROGET.

